# Determining Reference Ranges for Total T_4_ in Dried Blood Samples for Newborn Screening

**DOI:** 10.3390/ijns6010017

**Published:** 2020-03-04

**Authors:** Anna-Isabella Hijman, Daniel Konrad, Ralph Fingerhut

**Affiliations:** 1 University Children’s Hospital Zurich, 8032 Zurich, Switzerland; 2 Department of Endocrinology & Diabetology, Children’s Research Center, University Children’s Hospital Zurich, 8032 Zurich, Switzerland; 3 Swiss Newborn Screening Laboratory, Children’s Research Center, University Children’s Hospital Zurich, 8032 Zurich, Switzerland

**Keywords:** congenital hypothyroidism (CH), newborn screening, prematurity, total thyroxine (tT_4_), thyroid stimulating hormone (TSH), thyroxine stability, reference range, dried blood sample (DBS)

## Abstract

The purpose of this study was to define reference intervals for total thyroxine (tT_4_) in dried blood samples (DBSs) obtained for newborn screening. The aim of our study was to assess the possible benefit of measuring tT_4_ concentrations directly in DBSs obtained for newborn screening in premature and term-born infants. In order to have a sufficient number of samples for the extremely premature infants (<30 weeks), we set up a retrospective study, measuring the concentrations in DBSs collected over the previous 21 weeks. This time frame was a result of the included miniature study of tT_4_ stability in DBSs. We found that tT_4_ strongly correlated with gestational age (GA) in premature infants, highlighting the need for age-specific reference ranges. For term-born infants, the tT_4_ ranges did not vary significantly among different gestational ages, allowing for the use of one single reference range.

## 1. Introduction

Congenital hypothyroidism (CH) is one of the most common pathologies of the thyroid present at birth. It is also one of the most common causes of mental retardation, which is preventable if treated early. It occurs in approximately 1:2000 to 1:4000 newborns and presents almost no symptoms until weeks after birth, when most of the damage in the brain is irreversible [1]. For this reason, neonatal screening was started in the 1970s, including the determination of either thyroxine (T_4_) or thyroid stimulating hormone (TSH), to detect children with such a condition as early as possible [2].

For a deeper understanding of the present work and the values discussed in it, it is important to recall the physiology of the thyroid hormone system after birth in term and preterm infants. In the first 30 min after birth, TSH rises abruptly, as a consequence of both exposure to a colder environment and the clamping of the umbilical cord. After this initial peak, the serum TSH concentration decreases rapidly over the first 24 h post-partum. The concentration continues to fall in the following week, but at a much slower pace. 

This initial surge in TSH stimulates the production of T_4_, which presents a peak a little later at approximately 24–36 h after birth. The same is true for triiodothyronine (T_3_), which rises because of both the TSH and T_4_ peaks. Newborn screening (NBS) is usually performed after these initial changes. In Switzerland, this screening occurs between 72–96 h after birth. In the subsequent week, the concentrations of these hormones fall and then effectively stabilize a level that is slightly higher than that of adults [3]. 

Premature born infants undergo these same transitional changes, but their hypothalamic–pituitary–thyroid axis (HPT-axis) is still immature. Consequently, these changes are quantitatively much smaller and serum concentrations of thyroxin-binding globulin (TBG), tT_4_, fT_4_, and T_3_ are lower than in term-born infants [4]. As mentioned in the previous paragraph, T_4_ concentrations drop after the first peak at 72–96 h. In premature-born infants, this drop is greater due to a higher T_4_ clearance. Serums T_4_ and T_3_ concentrations then rise again after the first week, reaching concentrations comparable to those of term-born infants by three to six weeks of life. This difference appears to be more marked in very preterm infants (<33 weeks), while more mature healthy preterm infants (34–36 weeks) have T_4_ values comparable to those of term-born infants after the first week of life [4]. Other factors that could affect tT_4_ concentrations are sex, ethnicity, seasonality, age at sampling, and other factors, such as TBG and albumin.

It is important to distinguish the two main types of CH: primary CH (T-CH) and central CH (C-CH). Primary congenital hypothyroidism, also called thyroidal hypothyroidism, affects approximately 1:2000–1:4000 newborns [5]. It is caused by a lack of thyroid hormone production, namely T_4_ and T_3_, mostly due to a dysfunctional thyroid gland (thyroid dysgenesis or dyshormonogenesis). 

Secondary CH, also called hypopituitary CH or central CH, is not of thyroidal origin, but rather of pituitary origin. This means that the problem lies within the production of TSH. C-CH is a much rarer cause of hypothyroidism, with a prevalence of 1:20,000 to 1:80,000 in the general population [5,6]. Among infants with C-CH, the TSH values are more U-shaped, which means that TSH measurements can result in being either low, normal, or increased. Also, fT_4_ levels are usually lower in C-CH than in T-CH with comparable TSH values [7].

Understanding this distinction between T-CH and C-CH is important for understanding the rationale behind the various screening programs. The primary T_4_-follow up TSH screening effectively detects T-CH, some cases of C-CH, and a delayed TSH rise, but it misses cases of subclinical T-CH. The primary TSH screening detects T-CH and subclinical T-CH effectively but misses most cases of C-CH and a delayed TSH rise if just one specimen is tested [8].

Like most neonatal screening programs worldwide, Switzerland has TSH-based NBS. If the values measured in the NBS are abnormal or if a particular infant needs its values monitored, they get recalled for a second blood draw, in which TSH and total T_4_ gets checked. To date, there are no reference values for T_4_ concentration in DBSs in term- or in premature-born infants.

The main aim of this study was to determine a reference range of total T_4_ in DBSs for term- and premature-born infants. If we can define a reference range of total T_4_ at birth, we could potentially spare children with abnormal or borderline TSH values at the NBS a second visit at a clinic for blood drawing. Instead, we could directly measure this value from the DBSs taken for NBS and interpret it with the reference values according to the gestational age (GA) of the newborn.

To get a good idea of the reference ranges of tT_4_ in term and preterm newborns, there is a need for a big sample size due to the big variance of tT_4_ values depending on birth weight, gestational age, etc. For this, we initiated a miniature study to determine the stability of tT_4_ in our stored DBSs.

## 2. Materials and Methods

This was a cross-sectional, experimental study carried out at the newborn screening laboratory at the University Children’s Hospital Zurich. The experiment was divided into two main parts. For the first part of the study, we used dried blood samples (DBSs) where tT_4_ was already measured and remeasured these samples after 0–11 weeks to determine its stability. The second part was subdivided into two additional parts, preterm and term-born infants, where we used the data from the first part to determine which samples were still indicative of the T_4_ concentration at the NBS.

### 2.1. tT_4_ Stability in Dried Blood Samples

To determine the stability of tT_4_ in the DBSs, we compared values, which were measured soon after the blood was drawn from the infant, i.e., reliable tT_4_ concentrations, with the results of our measurements taken in the same week and up to multiple weeks after storage of the DBSs. The cards were stored at room temperature in a dark and dry room inside the newborn screening laboratory.

For every storage time span we wanted to check the stability for, we used 10 specimens. For the first batch of 10 cards, the first measurement was within the same week; for the second batch, the first measurement dated back 1 week, and so on. We used a time span interval of 1 week for up to 7 weeks of storage. After 7 weeks, we increased the time span interval to approximately 2 weeks, and then increased it again to 3 weeks between measurements (Table 1, see Results).

For some weeks, the desired minimum number of 10 DBSs was not available. The reason for this was, as mentioned earlier, that in Switzerland, we have a TSH-based screening; therefore, thyroxine was only measured after a first pathological or borderline value of TSH, resulting in relatively few measurements per week. 

As seen in Table 1 (see Results), we measured 10–11 samples for each of the storage times considered. For the storage times of 5, 19, and 21 weeks, we removed one outlier, leaving those groups consisting of only nine samples.

For this first part of the study, we measured the thyroxine values of a total of 255 specimens belonging to newborns with (borderline) pathological TSH measurements at the newborn screening. 

### 2.2. Determination of tT_4_ Ranges

#### 2.2.1. tT_4_ Values of Preterm-Born Infants

For the main part of the study, we selected all premature-born infants who were born in the preceding 21 weeks. This time span was determined through the results of the first part, in which we found that thyroxine could be considered stable, with some correction, over a maximum of 21 weeks. The correction was applied to all DBSs with a storage time of 36 to 146 days and amounted to 10%, i.e., a 10-week-old DBS with a measured thyroxine value of 95 nmol/L was corrected by adding 10%, resulting in an effective value of 104.5 nmol/L (see Results). 

**Figure 1:**
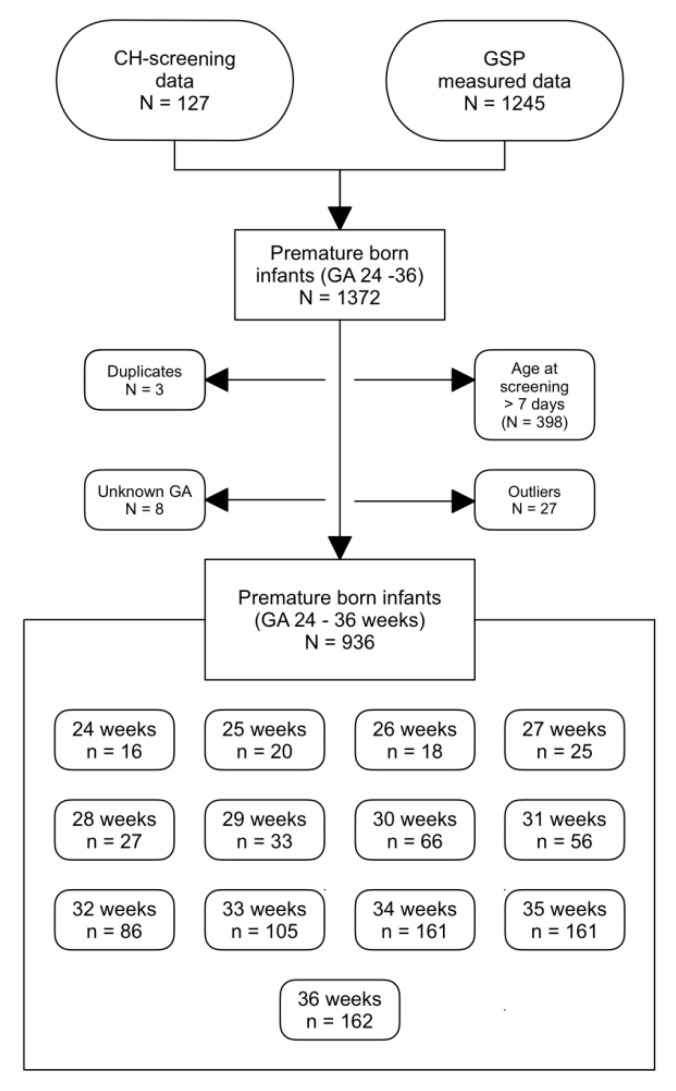
Data selection for the study. Outliers were determined using SPSS. GA: gestational age, GSP: GSP Neonatal Thyroxine (T4) kit.

In total, we measured 1245 dried blood samples of premature newborns at different gestational ages. We also included further 127 tT_4_ measurements, which were assessed outside of this experiment as part of a CH-screening. After excluding the measurements that were taken at >7 days of age, the duplicate values between the two data sets and the outlying values, our data set consisted of 944 DBSs of infants born at a gestational age between 24 and 36 weeks (Figure 1).

We adopted the World Health Organization (WHO) definition of premature, i.e., infants born before completion of the 37th pregnancy week. The most premature infants mentioned in this paper where born in the 24th pregnancy week. 

The specimens were measured using the GSP^®^ Neonatal Thyroxine (T4) kit by PerkinElmer (Turku, Finland).

#### 2.2.2. tT_4_ Concentrations of Term-Born Infants

The thyroxine values for the term-born infants were obtained the same way as the values of the premature-born infants. Because full-term-born infants were more prevalent than premature-born infants, we were able to measure the thyroxine values of 973 infants over the time it took to collect the measurements for the premature-born infants. These values were taken together with the regular newborn screening measurements; therefore, no correction for storage time had to be added. 

## 3. Results

### 3.1. Stability of tT_4_ in Dried Blood Samples

**Figure 2:**
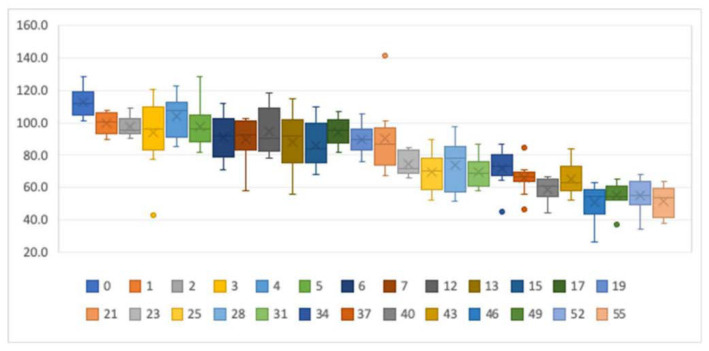
Boxplots showing the tT_4_ stability for different storage times. *y*-axis: percentage of initial tT4 concentrations. *x*-axis: storage time in weeks. The dots represent outliers.

Figure 2 shows the percentages of the post-storage tT_4_ concentrations relative to the baseline concentrations. Measurements taken in the same week (0) showed an increase of the tT_4_ concentration with the second measurement. There seemed to be a stability of the concentrations over the first 5 weeks. Storage times of 6–21 weeks seemed to remain stable at around 90% of the baseline value. After 21 weeks of storage, concentrations dropped drastically.

When we compared the baseline concentrations with the post-storage concentrations in a paired *t*-test, differences were not statistically significant between the measurements for up to 6 weeks of storage (see Table 1). In contrast, measurements taken in the same week were statistically different. On the other hand, measurements taken after 12, 13, and 17 weeks of storage were not statistically different between measurements. The differences between measurements displayed a normal distribution in all groups.

We therefore concluded that DBSs taken within the last 5 weeks can be considered to be 100% of the original value, whereas values for the specimens older than 6 weeks should be corrected by adding 10% to the measured valued. DBSs older than 21 weeks need to be discarded since they no longer reflect the true thyroxine concentration.

To check whether the corrected concentrations reflect baseline values, a paired *t*-test was applied for the corrected post-storage concentrations of samples stored for 6–21 weeks. As depicted in Table 2, the corrected post-storage values showed no significant difference compared to their correspondent baseline values for storage times between 6 and 21 weeks.

### 3.2. Determination of the tT_4_ Ranges

#### 3.2.1. tT_4_ Values of Preterm-Born Infants

**Figure 3:**
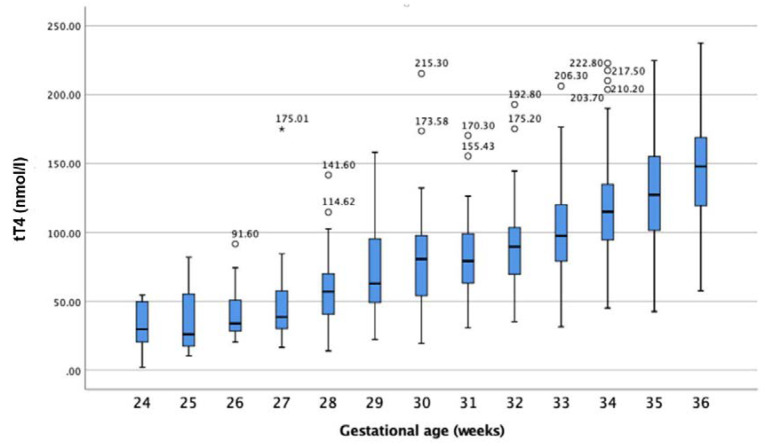
tT_4_ concentration (nmol/L) in relation to gestational age. Circles represent outliers, and stars extremes.

In premature infants, tT_4_ concentrations in DBSs were related to GA, as shown in Figure 3. Infants with a lower GA revealed lower tT_4_ concentrations than infants born with a higher GA (see Table 3). Overall, the tT_4_ values ranged from under 10 nmol/L in infants born in the 24th week of pregnancy to a maximum of 237.5 nmol/L in infants born in the 36th week of pregnancy. 

#### 3.2.2. tT_4_ Concentrations of Term-Born Infants

Thyroxine values were measured for 973 newborns. Seventeen values were measured at an age of >7 days, 10 measurements with no information about GA, and 78 measurements of premature born infants were excluded, resulting in 868 samples of term-born infants (GA 37–43 weeks).

**Figure 4:**
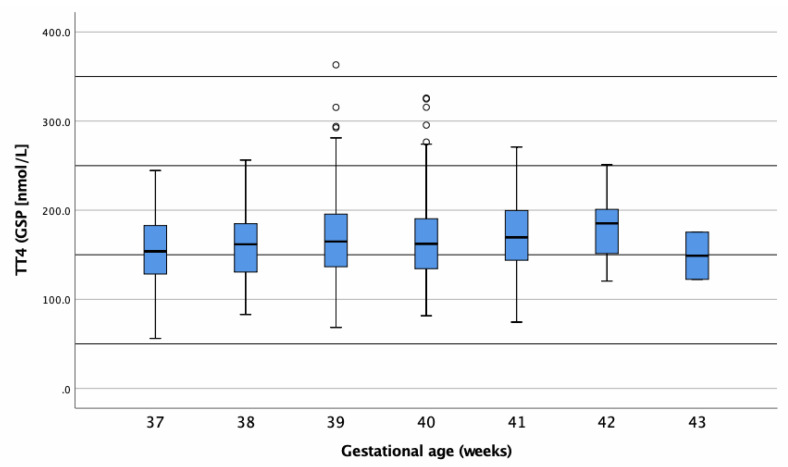
Boxplot of tT_4_ concentrations (nmol/L) in dried blood samples (DBSs) of term-born infants. Circles represent outliers.

Values presented a normal distribution with a mean value of 166.4 nmol/L (12.8 μg/dL) and a median value of 164.2 (12.6 μg/dL). As depicted in Table 4 and Figure 4, term born infants (≥37th pregnancy week) had tT_4_ values within a mean of approximately 150–180 nmol/L, independent of their GA at birth.

#### 3.2.3. tT_4_ Differences between Genders 

**Figure 5:**
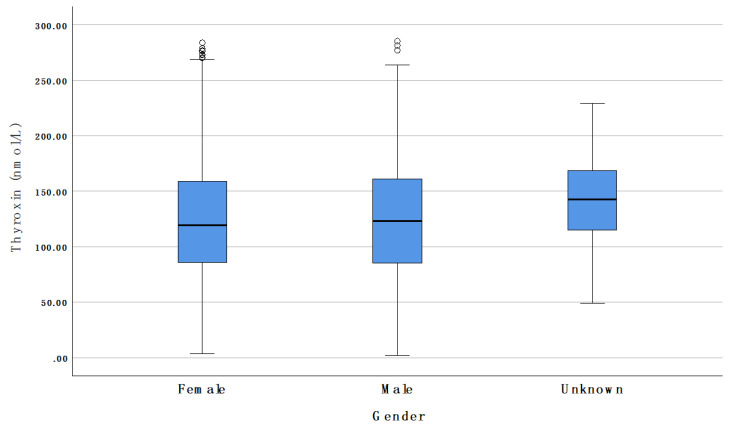
Comparison of tT_4_ concentrations between male and female newborns. Circles represent outliers.

As shown in Table 5 and Figure 5, there were no differences in tT_4_ when comparing male and female infants overall. The same was true when comparing male and female infants of the same gestational age.

### 3.3. Ethics

This study was approved by the cantonal ethics committee (BASEC-No.: 2019-01901/22 November 2019).

## 4. Discussion 

### 4.1. Thyroxine Stability Testing

Prematurely born infants account for only 7% [9] of the newborn infants in Switzerland, which amounts to approximately 6000 prematurely born infants each year. In order to have a valid reference population, we first determined the stability of thyroxine in the stored DBSs. We then set up a retrospective study using those screening cards, in which thyroxine was still comparable to its pre-storage value. 

When compared with the available literature on DBS storage, we found differing results. One study from Brazil found that thyroxine values could be considered reliable for further hormonal testing for at least 36 months; however, specimen stability was evaluated at 4–8 °C [10]. Another study reported that T_4_ is the most sensitive hormone amongst the thyroid-related hormones and remains stable for a week in DBSs; after that its value declines rapidly when stored at room temperature. However, this was a study from 1987 with different materials and methods of measurement. Also, their method of studying thyroxine stability included a control sample that was stored at −20 °C. Our values were directly compared with their relative measurements taken shortly after the blood samples were taken [11].

Baseline and relative post-storage values were compared using a paired samples *t*-test. As shown in Table 1 (see Results), post-storage measurements taken in the same week as their baseline measurements were significantly higher. This suggests that there was a significant measurement error due either to the measurement variability of the method used or to the small sample size.

Under the conditions mentioned in the methods section, it appears that there was no significant difference between the baseline and post-storage concentrations for up to 5–6 weeks. Our findings did not reflect previously reported findings, which stated that thyroxine values start decreasing significantly after four days of suboptimal storage conditions, including storage at room temperature. Davis et al. found that high temperature and humidity are especially detrimental for tT_4_ stability. Specimens should therefore be stored in sealed bags and kept in low temperature and low humidity conditions to ensure the hormone stability of DBSs [12].

### 4.2. Reference Intervals for Newborn and Premature-Born Infants

Child development and growth have a strong influence on the reference intervals of many biochemical markers [13]. As shown herein, tT_4_ seemed to be influenced by intrauterine development. As depicted in Figure 3, tT_4_ correlated with GA. Infants born at a more mature GA had higher concentrations of tT_4_. These findings suggest a need for GA-appropriate reference intervals for premature-born infants. Alternatively, as reported previously, tT_4_ also strongly correlates with birthweight (BW). It remains to be determined which of these two factors has a stronger impact on tT_4_. 

Few studies have tried to determine reference ranges for tT_4_ [14,15] and we found no studies measuring tT_4_ from DBSs. Total T_4_ reference ranges may be useful in premature-born infants when combined with standard TSH measurements. As reported by different studies [16,17], there seems to be a higher prevalence of delayed TSH increase in low-birthweight (LBW) and very low birthweight (VLBW) infants. Therefore Mandel et al. suggested an additional T_4_ measurement in LBW and VLBW infants with additional routine testing to avoid missing atypical hypothyroidism [18].

Premature-born infants will often have false-positive or false-negative results. False-positive results are a result of the typical fluctuation in thyroid hormones in the first few weeks of life. As we will discuss below, preterm infants often present with hypothyroxinaemia. The latter is usually mild and only transient but can cause a false-positive screening result if screening is based on thyroxine measurements with a secondary TSH measurement. As a result of such hypothyroxinaemia, these infants might also show a mild TSH rise, causing false positive results in TSH-based screening. Another problem with the TSH-based screening is the notion that it will also miss premature-born infants with T-CH, as they are more likely to have a delayed TSH rise (false negative) [19,20].

A TSH–T_4_ combined screening for premature-born infants could possibly detect C-CH and/or T-CH with a greater accuracy and earlier when appropriate reference ranges are used (according to GA instead of general newborn cut-off values) since tT_4_ levels of premature infants were significantly lower than in term-born infants, as demonstrated herein. However, more elaborate studies need to be done in order to establish a distinction between premature infants with transient hypothyroxinaemia (isolated low T_4_) and true C-CH (low T_4_ and low, normal or slightly elevated TSH).

### 4.3. Premature-Born Infants (24–36 Weeks)

#### 4.3.1. Reference Individuals

In general, direct sampling is preferred to indirect sampling because the latter may contain values that are influenced by eventual pre-existent diseases of the selected population. When a lot of values differ from physiological values, the reference interval is much less sensitive [20]. These are important considerations to keep in mind since our main sample population was premature born infants, who often have associated health problems. We must, however, also acknowledge the fact that obtaining analytical values from newborns, and especially from premature infants, is a very difficult task for multiple practical and ethical reasons. 

To obtain a big enough reference population for each of our groups, we measured tT_4_ concentrations from the original newborn screening cards. We separated term- and premature-born infants, and further separated premature infants according to their GA at birth. We had no information regarding the health status of the infants at the time of the newborn screening.

#### 4.3.2. Differences in Thyroid Hormone Regulation Post-Partum between Premature- and Term-Born Infants

The difference between premature and postnatal thyroid function is mainly quantitative, meaning that physiological changes that happen in term-born infants also happen in premature-born infants, only attenuated [4]. The more premature the infant the bigger the attenuation is. 

As shown in the results section (see Figure 3), the tT_4_ concentrations were proportional to GA. The lower the GA at birth, the smaller the concentration of tT_4_ found in the DBSs. Our results agree with Williams et al., who reported lower tT_4_ values in more premature infants. In their study, Williams et al. found slightly higher tT_4_ at the 7th day post-partum. However, their measurements were taken from serum and they grouped infants of different GAs together [21]. 

After an initial T_4_ peak at approximately 24 h post-partum, thyroxine concentrations of term-born infants decrease gradually over the first weeks of life [3]. Moreover, Williams et al. found that postnatal T_4_ increases were attenuated in slightly premature infants (31 to 34 weeks of gestation), absent in more premature infants (28–30 weeks of gestation), and even reversed in the most premature ones (23–27 weeks of gestation) [21]. This could explain some of the extremely low T_4_ concentrations we found in the lower GA groups as our measurements were obtained from DBSs taken in the first 3–7 days of life.

According to Chung et al., there is a high incidence of thyroid dysfunction in preterm infants. In premature born infants, the TSH surge and pituitary feedback for thyroid hormones are attenuated. Therefore, TSH may not be increased even when serum thyroid hormones are low. As such, TSH levels are not representative of overall thyroid function in premature born infants. Repeated thyroid screening tests including TSH and T_4_ in preterm infants may overcome these limitations [22].

#### 4.3.3. Reasons for Hypothyroxinaemia in Premature Infants

Transient hypothyroxinaemia is a common condition in infants born prematurely and/or with a low birthweight and is characterized by low levels of thyroid hormones with a normal TSH concentration. Consequently, it will often be missed when screening is based on TSH, even though it is found in approximately 50% of premature-born infants. 

Reasons for hypothyroxinaemia in premature infants are complex since there are multiple possible causes. The main factors are an immature hypothalamo–pituitary–thyroid (HPT) axis, low TBG levels, and decreased conversion of T_4_ to T_3_ [23]. Furthermore, very premature-born and/or VLBW infants often have accompanying systemic diseases and may be treated with drugs that interfere with the HPT-axis [22]. 

#### 4.3.4. Consequences of Hypothyroxinaemia in Premature-Born Infants

Multiple studies [24,25] have found correlations between low levels of T_4_ and negative outcomes, such as mortality and neurodevelopmental deficits later in life. The latter includes lower IQ, delayed psychomotor development, and increased risk of cerebral palsy [26]. These findings would suggest early detection and treatment to be beneficial and important for central nervous system (CNS) development. 

However free T_4_ (fT_4_), the biologically available thyroid hormone, does not correlate with GA or postnatal age beyond the first week of life. Furthermore, its levels stay relatively stable despite widely varying TSH concentrations and are similar to the concentrations found in adults. This would suggest that the serum fT_4_ concentration is closely regulated by the HPT axis despite varying TSH levels [27]. The latter is supported by more recent studies reporting no association between the hypothyroxinaemia of prematurity and neurodevelopmental outcome in young adulthood, in particular, no association with IQ score and motor function has been found [28]. A review article by La Gemma et al. highlighted the importance of thyroid hormones in CNS development, but also found no clear effect of T_4_ supplementation for transient hypothyroxinaemia of prematurity (THOP) [29]. Similarly, van Wassenaer et al. reported that thyroxine supplementation did not improve the developmental outcome at 24 months of age in infants born very prematurely (<30 weeks GA) [30]. Lastly, Carrascosa et al. studied thyroid function during the first year of life of 75 healthy premature born infants between 30 and 35 weeks GA. They found that these premature infants can adapt their post-natal response and meet the needed levels of thyroid-related hormones by the first few weeks after birth [4].

#### 4.3.5. Thyroxine Reference Ranges for Premature Infants

Our study revealed that the tT_4_ reference interval for premature-born infants was related to GA. Consequently, the reference intervals were lower than those of term-born infants and were divided by gestational age at birth. For the most premature infants, our study included only a few measurements because of the rareness of such premature births in our study population. For this reason, further studies should be done, not only to evaluate the importance of tT_4_ measurements in the DBSs for NBS, but also to define more accurate reference values for newborns born at a GA <30 weeks.

### 4.4. Term-Born Infants (37–43 Weeks)

#### 4.4.1. Reference Ranges for Term-Born Infants

As shown in Table 4, our study revealed a tT_4_ reference range of 83–250 nmol/L (6.4–19.2 µg/dL) for healthy, term-born infants. Such a reference range was defined as mean ± 2 SD (95% of healthy infants) due to the normal distribution of tT_4_ concentrations. There was no significant difference between male and female newborns (Figure 5 and Table 5). We found no other studies where tT_4_ concentration was measured in DBSs for NBS. Most studies measured either fT_4_, cord T_4_, or serum T_4_; therefore comparisons with these studies were not possible [22,31].

#### 4.4.2. Central Congenital Hypothyroidism

Multiple studies underline the importance of C-CH detection by arguing that C-CH is more common than previously thought [4,32]. Also, according to Zwaveling-Soonawala et al., C-CH may fulfil the screening criteria because it is relatively frequent, testing methods are inexpensive, effective treatment is available, and the risks of an unfavorable outcome are well known [33]. However, to date, there are no studies reporting a benefit from early detection during newborn screening as opposed to later detection from clinical presentation.

Countries with combined T_4_ and TSH (and TBG) determination have the benefit of also diagnosing cases of C-CH, despite a slightly less sensitive diagnosis of T-CH. Lanting et al. concluded that T_4_ plus TSH and TBG is an effective method for detecting C-CH and preventing severe morbidity in affected newborns. They also argue that the costs of adding TBG to a T_4_ with a reflectory TSH method are acceptable, especially when one considers the costs of long-term morbidity as a consequence of late detection of the disease [34].

### 4.5. Limitations 

The limitations of this study included a relatively small sample size for our thyroxine stability experiment. The small sample size may reduce the accuracy of included measurements since we found no clear delineation between stabilities at different storage times. We found no significant difference between the corrected post-storage values and their baseline, indicating a fairly good cut-off at 6 weeks of storage time. However, a bigger sample size would yield a more accurate determination of thyroxine stability in DBSs. 

Another limitation was the storage conditions of the DBS. The available DBSs included in our study were stored in a small and dry room inside of the laboratory for newborn screening. However, there was no strict control for humidity or room temperature, and the DBSs were not kept in sealed plastic bags, as suggested by Davis et al. [12].

To increase our sample size of newborn infants, we included measurements taken as part of a CH screening after an abnormal or borderline TSH measurement. Even though none of the infants had CH, abnormal TSH levels at birth might have influenced the tT_4_ values in their DBSs. However, direct comparison of tT_4_ concentrations in both groups with GA did not show significant differences between the two data sets. As outlined above, only a small number of preterm babies with lower GA were included. Therefore, one high concentration could have increased the reference range by a significant degree.

As infants born at less than 30 weeks of GA are quite uncommon, we had a small sample size for infants between 24 and 30 weeks. Nevertheless, we chose to keep these infants in separate groups given that even in small sample sizes, one can see a lower tT_4_ concentration with lower GAs. We acknowledge, however, that more studies with bigger sample sizes need to be done in order to confirm our findings.

## 5. Conclusions

We suggest separate reference values for preterm- and term-born infants. However, before clinical use of tT_4_ measurements of DBSs is appropriate for NBS, more studies with bigger sample populations are needed. Whenever possible, the sample size should be determined in advance and measurements taken directly at the NBS, eliminating measurement errors due to DBS storage. Also, future studies should eliminate as many confounders as possible since there are many of them that possibly influencing tT_4_ values, especially in preterm infants, who often have other health conditions associated with premature birth.

## Figures and Tables

**Table 1:** Mean thyroxine baseline and post-storage concentrations.

Storage Time	*n*	tT_4_ (nmol/L)				*p*-Value
		Baseline		Post Storage		
		Mean	95% CI	Mean	95% CI	
0 weeks	10	116.0	88.15–143.98	128.6	102.2–155.06	0.000
1 week	10	108.4	87.25–129.63	106.9	89.88–123.90	0.572
2 weeks	10	128.1	109.35–146.85	124.2	107.08–141.36	0.136
3 weeks	10	114.4	90.52–138.36	106.9	81.64–132.08	0.345
4 weeks	10	109.5	94.31–124.71	113.8	95.74–131.88	0.346
5 weeks	9	110.6	84.02–137.23	104.0	79.66–128.34	0.075
6 weeks	10	113.8	89.94–137.56	100.5	87.74–113.22	0.059
7 weeks	11	111.2	87.47–134.93	98.4	74.66–122.20	0.020
12 weeks	10	105.1	92.24–117.90	99.4	84.53–114.21	0.287
13 weeks	10	103.4	86.76–119.96	90.2	72.90–107.40	0.068
15 weeks	10	112.0	95.03–128.87	95.9	81.13–110.61	0.025
17 weeks	10	108.9	95.30–122.56	103.0	86.46–119.58	0.084
19 weeks	9	118.4	102.01–134.77	108.9	90.08–127.81	0.007
21 weeks	9	106.5	86.17–126.90	91.3	67.76–114.80	0.003
23 weeks	10	134.4	108.22–160.60	99.3	81.78–116.82	0.000
25 weeks	11	118.1	96.31–139.91	82.4	61.34–103.50	0.000

**Table 2:** Mean thyroxine concentrations at baseline and corrected by 10% after storage.

Storage Time	*n*	tT_4_ (nmol/L)				*p*-Value
		Baseline		Post Storage and Post Correction		
		Mean	95% CI	Mean	95% CI	
6 weeks	10	113.8	89.94–137.56	110.53	96.51–124.54	0.594
7 weeks	11	111.2	87.47–134.93	108.27	82.12–134.41	0.570
12 weeks	10	105.1	92.24–117.90	109.31	92.98–125.63	0.455
13 weeks	10	103.4	86.76–119.96	99.16	80.18–118.14	0.551
15 weeks	10	112.0	95.03–128.87	105.46	89.25–121.67	0.324
17 weeks	10	108.9	95.30–122.56	113.32	95.11–131.54	0.245
19 weeks	9	118.4	102.01–134.77	119.84	103.49–143.31	0.660
21 weeks	9	106.5	86.17–126.90	100.41	78.91–124.78	0.181
23 weeks	10	134.4	108.22–160.60	109.23	89.96–128.50	0.000
25 weeks	11	118.1	96.31–139.91	90.66	67.47–113.85	0.000

A paired *t*-test was used to analyze differences between the baseline and after storage concentrations.

**Table 3:** Mean tT_4_ concentration and percentiles for premature-born infants.

GA at Birth (Weeks)	Number Studied (*n*)	Mean ± SD	Percentiles or (Min–Max) *		Unit
			2.5	97.5	
24	16	32.52 ± 17.1	(1.98–54.45)		nmol/L
		2.5 ± 1.3	(0.15–4.23)		µg/dL
25	20	39.7 ± 25.8	(10.50–87.30)		nmol/L
		3.1 ± 2.0	(0.81–6.78)		µg/dL
26	18	36.7 ± 16.7	(15.40–74.30		nmol/L
		2.8 ± 1.3	(1.19–5.77)		µg/dL
27	25	43.2 ± 17.1	(16.60–84.48)		nmol/L
		3.3 ± 1.3	(1.29–6.56)		µg/dL
28	27	57.9 ± 24.8	(15.40–114.62)		nmol/L
		4.4 ± 1.9	(1.20–8.90)		µg/dL
29	33	76.6 ± 37.6	(22.33–158.18)		nmol/L
		5.9 ± 2.9	(1.73–12.29)		µg/dL
30	66	77.6 ± 32.9	11.90	161.43	nmol/L
		6.0 ± 2.5	0.92	12.51	µg/dL
31	56	82.0 ± 31.3	33.52	163.98	nmol/L
		6.3 ± 2.4	2.60	12.71	µg/dL
32	86	86.1 ± 24.5	43.97	142.40	nmol/L
		6.6 ± 1.9	3.41	11.04	µg/dL
33	105	102.4 ± 32.6	44.44	172.57	nmol/L
		7.9 ± 2.5	3.44	13.38	µg/dL
34	161	115.5 ± 29.2	64.12	179.63	nmol/L
		8.9 ± 2.5	4.97	13.92	µg/dL
35	161	129.9 ± 39.5	52.26	211.16	nmol/L
		10.0 ± 3.0	4.05	16.37	µg/dL
36	162	144.4 ± 36.8	72.00	215.19	nmol/L
		11.1 ± 2.8	5.58	16.68	µg/dL

* For groups with *n* < 50, the lowest and highest tT_4_ value is given in brackets (min–max).

**Table 4:** Mean concentration of tT_4_ and percentiles according to gestational age.

GA at Birth (Weeks)	Number Studied (n)	Mean ± SD	Percentiles or (Min–Max) *		Unit
			2.5	97.5	
37	60	157.6 ± 39.9	81.09	*232.95*	nmol/L
		12.1 ± 3.1	6.30	18.10	µg/dL
38	155	162.1 ± 36.5	101.94	237.86	nmol/L
		12.5 ± 2.8	7.92	18.48	µg/dL
39	223	168.5 ± 44.2	95.80	269.42	nmol/L
		13.0 ± 3.4	7.44	20.93	µg/dL
40	260	156.9 ± 44.1	95.32	266.66	nmol/L
		12.8 ± 3.4	7.40	20.72	µg/dL
41	147	170.3 ± 39.3	94.94	242.85	nmol/L
		13.1 ± 3.0	7.37	18.87	µg/dL
42	21	182.7 ± 37.6	(120.50–250.90)		nmol/L
		14.1 ± 2.9	(9.36–19.49)		µg/dL
43	2	148.9 ± 37.5	(122.40–175.40)		nmol/L
		11.5 ± 2.9	(9.51–13.63)		µg/dL
All	868	166.4 ± 41.7	95.91	256.00	nmol/L
		12.8 ± 3.2	7.45	19.90	µg/dL

* For groups with *n* < 50, the lowest and highest value are given in brackets (min–max) measured in nmol/L.

**Table 5:** Comparison of tT_4_ concentrations between male and female newborns.

Gender	Number Studied (*n*)	Mean	Standard Deviation
Male	749	124.4	52.75
Female	619	123.7	55.10
Unknown	19	137.8	46.7

## Abbreviations

DBS	Dried blood sample
GA	Gestational age
T_3_	Triiodothyronine
T_4_	Thyroxine
tT_4_	Total thyroxine
fT_4_	Free thyroxine
TSH	Thyroid stimulating hormone
THOP	Transitory hypothyroxinaemia of prematurity
LBW	Low birthweight
VLBW	Very low birthweight
